# Na_7_Cr_4_(P_2_O_7_)_4_PO_4_

**DOI:** 10.1107/S1600536813008726

**Published:** 2013-04-10

**Authors:** Noura Bourguiba Fakhar, Mohamed Faouzi Zid, Ahmed Driss

**Affiliations:** a Laboratoire de Matériaux et Cristallochimie, Faculté des Sciences de Tunis, Université de Tunis El Manar, 2092 Manar II Tunis, Tunisia

## Abstract

The title compound, hepta­sodium tetra­chromium(III) tetra­kis­(diphosphate) orthophosphate, was synthesized by solid-state reaction. Its structure is isotypic with that of Na_7_ *M*_4_(P_2_O_7_)_4_PO_4_ (*M* = In, Al) compounds and is made up from a three-dimensional [(CrP_2_O_7_)_4_PO_4_]^7−^ framework with channels running along [001]. The three Na^+^ cations are located in the voids of the framework. One of the cations is situated on a general position, one is equally disordered around a twofold rotation axis and one is on a fourfold rotoinversion axis. The isolated PO_4_ tetra­hedron of the anionic framework is also situated on the -4 axis. Structural relationships between the title compound and different diphosphates containing *M*P_2_O_11_ units (*M* = Mo, V) are discussed.

## Related literature

For isotopic compounds, see: Stus *et al.* (2001 ▶); Zhao (2011 ▶). For background to physico-chemical properties of related compouds, see: Sljukic *et al.* (1967 ▶); Hagman & Kierkegaard (1968 ▶); Goodenough *et al.* (1976 ▶); Sanz *et al.* (1999 ▶); De la Rochère *et al.* (1985 ▶). For bond lengths and angles in related structures, see: Wang & Hwu (1991 ▶); Kouass *et al.* (2010 ▶). For structural relationships, see: Zid *et al.* (2003 ▶); Averbuch-Pouchot (1988 ▶); Benhamada *et al.* (1992 ▶); Hwu *et al.* (1994 ▶); Capitelli *et al.* (2007 ▶); Bohaty *et al.* (1982 ▶); Leclaire *et al.* (1988 ▶, 1989 ▶); Moya-Pizarro *et al.* (1984 ▶). For bond-valence param­eters, see: Brown & Altermatt (1985 ▶).

## Experimental

### 

#### Crystal data

Na_7_Cr_4_(P_2_O_7_)_4_PO_4_
*M* *_r_* = 1159.66
Tetragonal, 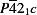

*a* = 14.058 (2) Å
*c* = 6.3103 (8) Å
*V* = 1247.1 (3) Å^3^
*Z* = 2
Mo *K*α radiation
μ = 2.54 mm^−1^
*T* = 298 K
0.24 × 0.16 × 0.11 mm

#### Data collection

Enraf–Nonius CAD-4 diffractometer
Absorption correction: ψ scan (North *et al.*, 1968 ▶) *T*_min_ = 0.61, *T*_max_ = 0.75
3276 measured reflections
1349 independent reflections
1277 reflections with *I* > 2σ(*I*)
*R*_int_ = 0.030
2 standard reflections every 120 min intensity decay: 1.1%

#### Refinement

*R*[*F* ^2^ > 2σ(*F* ^2^)] = 0.023
*wR*(*F* ^2^) = 0.060
*S* = 1.12
1349 reflections
123 parameters
Δρ_max_ = 0.30 e Å^−3^
Δρ_min_ = −0.26 e Å^−3^
Absolute structure: Flack (1983 ▶), 1349 Friedel pairs
Flack parameter: −0.03 (2)

### 

Data collection: *CAD-4 EXPRESS* (Duisenberg, 1992 ▶; Macíček & Yordanov, 1992 ▶); cell refinement: *CAD-4 EXPRESS*; data reduction: *XCAD4* (Harms & Wocadlo, 1995 ▶); program(s) used to solve structure: *SHELXS97* (Sheldrick, 2008 ▶); program(s) used to refine structure: *SHELXL97* (Sheldrick, 2008 ▶); molecular graphics: *DIAMOND* (Brandenburg, 1998 ▶); software used to prepare material for publication: *WinGX* (Farrugia, 2012) ▶.

## Supplementary Material

Crystal structure: contains datablock(s) I, global. DOI: 10.1107/S1600536813008726/wm2734sup1.cif

Structure factors: contains datablock(s) I. DOI: 10.1107/S1600536813008726/wm2734Isup2.hkl

Additional supplementary materials:  crystallographic information; 3D view; checkCIF report

## Figures and Tables

**Table 1:** Selected bond lengths (Å)

Cr1—O6	1.965 (2)
Cr1—O1^i^	1.967 (2)
Cr1—O8	1.972 (2)
Cr1—O3	1.973 (2)
Cr1—O2	1.977 (2)
Cr1—O4	1.993 (2)
P1—O6^ii^	1.496 (2)
P1—O1	1.515 (2)
P1—O4	1.518 (2)
P1—O7	1.593 (3)
P2—O5	1.496 (2)
P2—O2^iii^	1.514 (2)
P2—O3	1.523 (2)
P2—O7^iv^	1.620 (2)
P3—O8	1.545 (2)

Symmetry codes: (i) 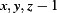; (ii) 
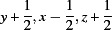
; (iii) 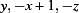; (iv) 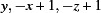.

**Fig. 1.**
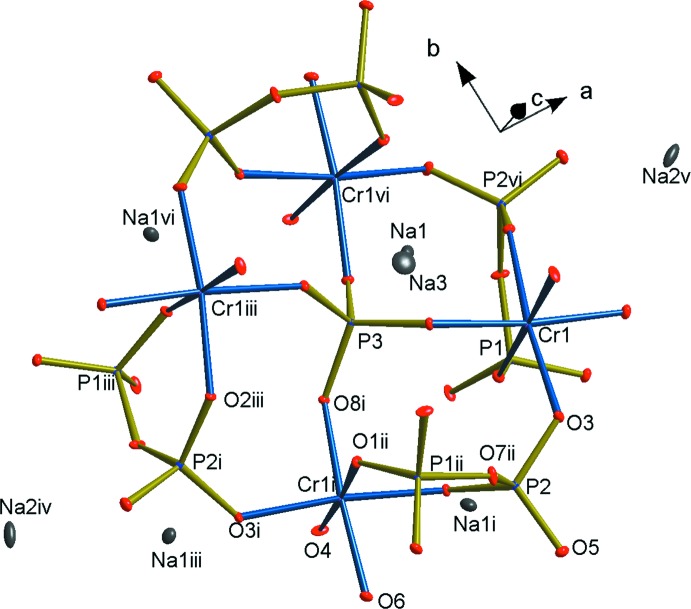
Part de la structure de Na7Cr4(P2O7)4PO4. Les éllipsoïdes ont été définis avec 50% de probabilité. [Code de symétrie: (i) y, 1 - x, 1 - z; (ii) y, 1 - x, 2 - z; (iii) 1 - x, 1 - y, 1 + z; (iv) -x + 1/2, y + 1/2, -z + 3/2; (v) 1 + y, -x + 1, -z + 2; (vi) -y + 1, x, -z + 1.

**Fig. 2.**
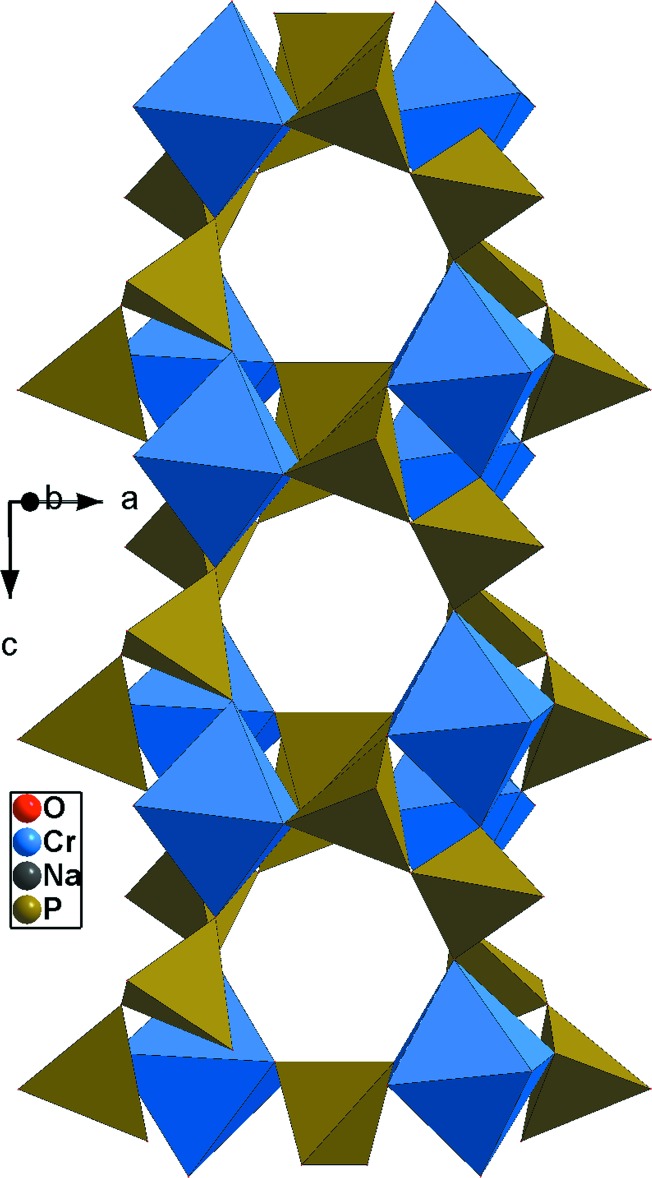
Représentation des colonnes selon c, montrant les connections entre les unités cycliques [CrP2O11].

**Fig. 3.**
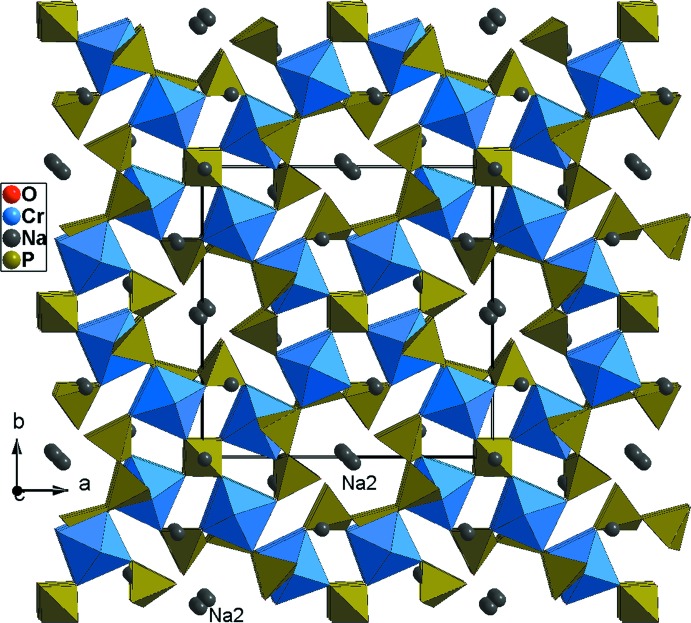
Projection de la structure de Na7Cr4(P2O7)4PO4, selon c montrant les canaux où résident les cations Na2.

**Fig. 4.**
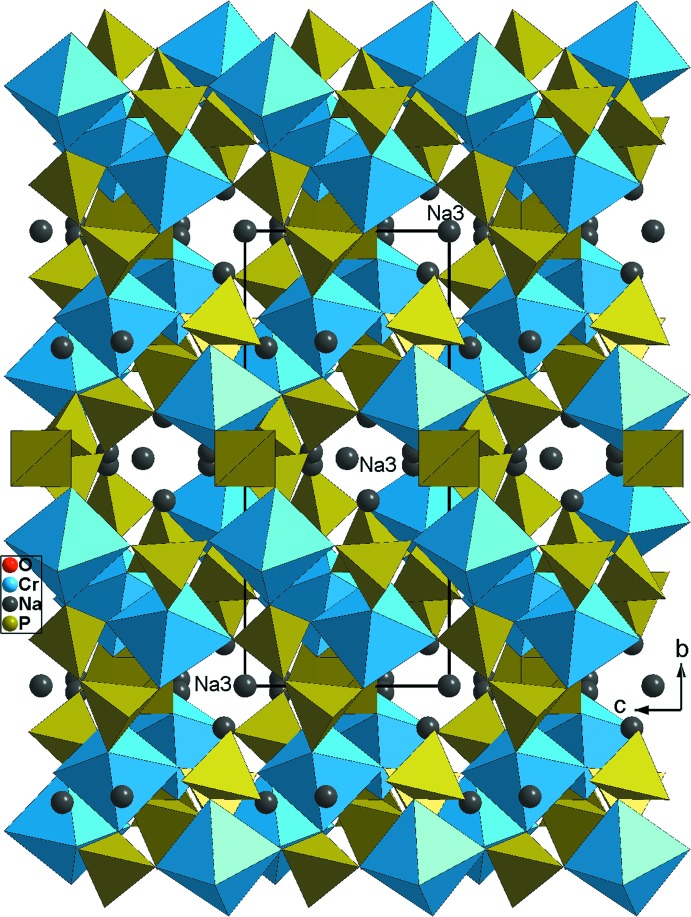
Projection de la structure de Na7Cr4(P2O7)4PO4, selon a, montrant des fenêtres étroites en face desquelles sont placés les cations Na3.

## supplementary crystallographic information

### Comment

Les dispositifs de stockage d'énergie comme les batteries, connaissent un essor important dans l'industrie, en particulier dans le domaine de la conversion des énergies renouvelables en énergie électrique. Cela a conduit à l'élaboration de nouveaux électrolytes solides ainsi qu'au développement des thématiques de recherche concernant la mobilité ionique au sein des matériaux cristallisés, notamment la famille NASICON: NaZr_2_(PO_4_)_3_ (Sljukic *et al.*, 1967; Hagman & Kierkegaard, 1968), Na_1+_*_x_*Zr_2_Si*_x_*P_3-_*_x_*O_12_ (Goodenough *et al.*, 1976) et un nouvel conducteur ionique à structure en couches Na_2_CoP_2_O_7_ (Sanz *et al.*, 1999). Une autre fammille de composés Na_7_*M*_4_(P_2_O_7_)_4_PO_4_ avec (*M* = Fe, Al, Cr) fût aussi découverte par De la Rochère *et al.* (1985), leurs propriétés d'échange ionique et de conductivité ont été étudiées. Dans cette série les composés Na_7_In_4_(P_2_O_7_)_4_PO_4_ et Na_7_Al_4_(P_2_O_7_)_4_PO_4_ ont été synthétisés et étudiés respectivement par Stus *et al.* (2001) et Zhao (2011). Cependant, pour le composé Na_7_Cr_4_(P_2_O_7_)_4_PO_4_, aucune analyse structurale n'a été signalée jusqu'ici.

La structure du composé Na_7_Cr_4_(P_2_O_7_)_4_PO_4_, admet une charpente tridimensionnelle constituée d'octaèdres CrO_6_, de tétraèdres PO_4_ et de groupements P_2_O_7_ reliés par partage de sommets. Les ions Na^+^ occupent les sites interstitiels. L'unité [(CrP_2_O_7_)_4_PO_4_]^7-^, de symétrie 4 inverse, est constituée par un tétraèdre central P3O_4_ dont chacun de ses quatre sommets oxygène O8 est lié à un octaèdre CrO_6_ appartenant respectivement à quatre unités [CrP_2_O_11_] différentes (Fig. 1). Dans ces dernières un groupement diphosphate P_2_O_7_ partage deux de ses sommets avec le même octaèdre CrO_6_. Dans cette structure, les unités cycliques [(CrP_2_O_7_)_4_PO_4_], (Fig. 1), se lient entre elles de part et d'autres, par formation de ponts mixtes de types Cr–O–P et P–O–Cr respectivement, entre les octaèdres CrO_6_ d'une unité et les groupements diphosphates P_2_O_7_ d'une autre unité, pour conduire à des colonnes infinies (Fig. 2) disposées selon la direction [001]. Remarquons que dans la charpente anionique, chaque colonne se lie à quatre voisines par partage de sommets entre polyèdres de nature différente pour conduire à une structure tridimensionnelle (Fig. 3) possèdant de larges canaux, à section hexagonale, parallèlles à la direction [001] où logent les cations Na2. La projection de la structure selon *a*, montre que les ions Na3 se placent en face de fenêtres étroites (Fig. 4). Il est à signaler que les cations Na^+^ sont distribués sur trois types de sites dans les interstices du réseau anionique. Deux de ces sites sont totalement occupés respectivement par Na1 en position générale (8*e*) et Na3 en position particulière (2*a*), quant au troisième site, il est partiellement occupé par le cation Na2 en position générale (8*e*), situé très proche de la position spéciale (4*d*). Les nombres de coordination des cations Na1 et Na2 sont de huit et cinq respectivement. Le cation Na3 se trouve au centre d'un tétraèdre régulier avec des longueurs de liaison toutes égales à 2,542 (2) Å. Le tétraèdre P3O_4_ est régulier, les longueurs de liaison P—O sont identiques et égales à 1,545 (2) Å. La géométrie des groupements P_2_O_7_ est conforme à celle observée dans de nombreux autres diphosphates (Wang & Hwu, 1991; Kouass *et al.*, 2010). Les distances interatomiques P—O varient globalement de 1,496 (2) Å à 1,622 (2) Å. Dans chacun de ces tétraèdres, on relève une distance longue pour l'oxygène du pont P1–O–P2 et une distance plus courte qui correspond aux atomes d'oxygène non reliés par ailleurs. Le calcul des différentes valences des liaisons utilisant la formule empirique (Brown & Altermatt, 1985) vérifie bien les valeurs (v.u.) de charges des ions: Cr1 (3,01), P1 (5,06), P2 (5,01), P3 (4,91), Na1 (1,12), Na2 (1,10) et Na3 (0,79) attendues dans la phase étudiée. La comparaison de la structure étudiée avec des travaux antérieurs et renfermant l'unité cyclique *M*P_2_O_11_ (*M* = Mo, V, Cr) révèle une certaine filiation entre la série de composés de formulation générale *AM*P_2_O_7_ (*A* = alcalin et *M* = métal trivalent), celles de *A*_2_MoO_2_P_2_O_7_ (*A* = K, NH_4_) et les deux phases Na_2_VOP_2_O_7_ et Li_9_(CrP_2_O_7_)_3_(PO_4_)_2_. En effet, les unités MoP_2_O_11_ se connectent par partage de sommets pour conduire à des rubans dans les structures unidimensionnelles de K_2_MoO_2_P_2_O_7_ (Zid *et al.*, 2003) et (NH_4_)_2_MoO_2_P_2_O_7_ (Averbuch-Pouchot, 1988). Ces unités cycliques [CrP_2_O_11_] se lient moyennant des tétraèdres PO_4_ pour conduire à des couches dans la structure de Li_9_(CrP_2_O_7_)_3_(PO_4_)_2_ (Capitelli *et al.*, 2007) et celles de type [VP_2_O_11_] se regroupent par partage de sommets entre octaèdres VO_6_ et tétraèdres PO_4_ des groupements diphosphates dans la structure bidimensionnelle de Na_2_VOP_2_O_7_ (Benhamada *et al.*, 1992). Elles sont liées par formation de ponts mixtes M–O–P dans les trois directions de l'espace pour former des charpentes tridimensionnelles dans la série de composés de formulation *AM*P_2_O_7_: β-NaTiP_2_O_7_ (Leclaire *et al.*, 1988), KMoP_2_O_7_ (Leclaire *et al.*, 1989), NaCrP_2_O_7_ (Bohaty *et al.*, 1982) et NaFeP_2_O_7_ (Moya-Pizarro *et al.*, 1984) ou bien dans la variété béta de BaV_2_(P_2_O_7_)_2_ (Hwu *et al.*, 1994). Afin d'utiliser ces données structurales et les relier aux propriétés physico-chimiques, en particulier de conduction ionique, et dès l'obtention d'une phase pure du composé étudié de formulation Na_7_Cr_4_(P_2_O_7_)_4_PO_4_ des mesures électriques moyennant un pont d'impédance complexe de type HP4192A seront réalisées.

### Experimental

La synthèse a été faite par voie sèche à partir d'un mélange de NH_4_H_2_PO_4_ (Scharlau, 62943), NaHCO_3_ (Prolabo, 27778) et CrCl_3_^.^6H_2_O (Merck, 2487), pris dans les proportion molaires Na:P:Cr = 8:3:9. Le traitement thermique a été réalisé en deux étapes: la première consiste à chauffer les réactifs à 673 K pendant 24 heures en vue d'éliminer les composés volatils. Après refroidissement et broyage, le mélange est porté à une température proche de sa fusion, 1023 K. Il est abandonné à cette température pendant une semaine pour favoriser la germination et la croissance des cristaux. Le résidu final a subi en premier lieu un refroidissement lent (5 K h^-1^) jusqu'à 923 K puis un second rapide (50 K h^-1^) jusqu'à l'ambiante. Des cristaux verts sous forme de parallélépipèdes, de taille suffisante ont été récupérés du flux par des lavages successifs à l'eau chaude. Un monocristal de contour net a été choisi sous microscope polarisant pour les mesures des intensités diffractées.

### Refinement

Les densités d'électrons maximum et minimum restants dans la Fourier-différence sont acceptables et sont situées respectivements à 1,45 Å de l'atome d'oxygène O8 et à 0,89 Å de l'atome de phosphore P2.

### Crystal data

**Table:**

Na_7_Cr_4_(P_2_O_7_)_4_PO_4_	*D*_x_ = 3.088 Mg m^−^^3^
*M**_r_* = 1159.66	Mo *K*α radiation, λ = 0.71073 Å
Tetragonal, *P*42_1_*c*	Cell parameters from 25 reflections
Hall symbol: P -4 2 n	θ = 11–15°
*a* = 14.058 (2) Å	µ = 2.54 mm^−^^1^
*c* = 6.3103 (8) Å	*T* = 298 K
*V* = 1247.1 (3) Å^3^	Prism, green
*Z* = 2	0.24 × 0.16 × 0.11 mm
*F*(000) = 1128	

### Data collection

**Table:**

Enraf–Nonius CAD-4 diffractometer	1277 reflections with *I* > 2σ(*I*)
Radiation source: fine-focus sealed tube	*R*_int_ = 0.030
Graphite monochromator	θ_max_ = 27.0°, θ_min_ = 2.1°
ω/2θ scans	*h* = −1→17
Absorption correction: ψ scan (North *et al.*, 1968)	*k* = −1→17
*T*_min_ = 0.61, *T*_max_ = 0.75	*l* = −8→8
3276 measured reflections	2 standard reflections every 120 min
1349 independent reflections	intensity decay: 1.1%

### Refinement

**Table:**

Refinement on *F*^2^	Secondary atom site location: difference Fourier map
Least-squares matrix: full	*w* = 1/[σ^2^(*F*_o_^2^) + (0.0275*P*)^2^ + 0.8748*P*] where *P* = (*F*_o_^2^ + 2*F*_c_^2^)/3
*R*[*F*^2^ > 2σ(*F*^2^)] = 0.023	(Δ/σ)_max_ = 0.001
*wR*(*F*^2^) = 0.060	Δρ_max_ = 0.30 e Å^−^^3^
*S* = 1.12	Δρ_min_ = −0.26 e Å^−^^3^
1349 reflections	Extinction correction: *SHELXL97* (Sheldrick, 2008), Fc^*^=kFc[1+0.001xFc^2^λ^3^/sin(2θ)]^-1/4^
123 parameters	Extinction coefficient: 0.0014 (4)
0 restraints	Absolute structure: Flack (1983), 1349 Friedel pairs
Primary atom site location: structure-invariant direct methods	Flack parameter: −0.03 (2)

### Special details

**Table:**

Geometry. All e.s.d.'s (except the e.s.d. in the dihedral angle between two l.s. planes) are estimated using the full covariance matrix. The cell e.s.d.'s are taken into account individually in the estimation of e.s.d.'s in distances, angles and torsion angles; correlations between e.s.d.'s in cell parameters are only used when they are defined by crystal symmetry. An approximate (isotropic) treatment of cell e.s.d.'s is used for estimating e.s.d.'s involving l.s. planes.
Refinement. Refinement of *F*^2^ against ALL reflections. The weighted *R*-factor *wR* and goodness of fit *S* are based on *F*^2^, conventional *R*-factors *R* are based on *F*, with *F* set to zero for negative *F*^2^. The threshold expression of *F*^2^ > σ(*F*^2^) is used only for calculating *R*-factors(gt) *etc*. and is not relevant to the choice of reflections for refinement. *R*-factors based on *F*^2^ are statistically about twice as large as those based on *F*, and *R*- factors based on ALL data will be even larger.

### Fractional atomic coordinates and isotropic or equivalent isotropic displacement parameters (Å^2^)

**Table:**

	*x*	*y*	*z*	*U*_iso_*/*U*_eq_	Occ. (<1)
Cr1	0.69014 (3)	0.38036 (3)	0.12660 (8)	0.00602 (13)	
P1	0.74875 (6)	0.38494 (6)	0.64161 (12)	0.00652 (18)	
P2	0.55451 (6)	0.20026 (6)	0.10207 (12)	0.00729 (17)	
P3	0.5000	0.5000	0.0000	0.0061 (3)	
Na1	0.75651 (11)	0.59258 (11)	0.3964 (2)	0.0183 (3)	
Na2	0.5141 (6)	−0.0143 (7)	0.8247 (4)	0.0306 (19)	0.50
Na3	0.5000	0.5000	0.5000	0.0371 (9)	
O1	0.68067 (16)	0.35965 (16)	0.8190 (3)	0.0105 (5)	
O2	0.74924 (16)	0.50631 (17)	0.0806 (3)	0.0093 (4)	
O3	0.64622 (16)	0.24863 (16)	0.1722 (4)	0.0126 (5)	
O4	0.69139 (17)	0.39839 (19)	0.4399 (3)	0.0147 (5)	
O5	0.56956 (18)	0.09593 (15)	0.0691 (4)	0.0151 (5)	
O6	0.82198 (16)	0.33400 (16)	0.1196 (4)	0.0129 (5)	
O7	0.79078 (18)	0.48798 (18)	0.6892 (4)	0.0156 (6)	
O8	0.56314 (15)	0.43921 (15)	0.1477 (3)	0.0098 (4)	

### Atomic displacement parameters (Å^2^)

**Table:**

	*U*^11^	*U*^22^	*U*^33^	*U*^12^	*U*^13^	*U*^23^
Cr1	0.0069 (2)	0.0075 (2)	0.0037 (2)	0.0007 (2)	0.00016 (19)	0.00024 (19)
P1	0.0083 (3)	0.0072 (4)	0.0040 (3)	0.0003 (3)	−0.0001 (3)	−0.0005 (3)
P2	0.0069 (4)	0.0080 (4)	0.0070 (3)	0.0005 (3)	0.0005 (3)	0.0005 (3)
P3	0.0065 (4)	0.0065 (4)	0.0052 (7)	0.000	0.000	0.000
Na1	0.0212 (7)	0.0191 (7)	0.0145 (7)	0.0042 (6)	−0.0030 (6)	−0.0006 (6)
Na2	0.030 (5)	0.051 (6)	0.0106 (11)	−0.026 (3)	0.0022 (15)	−0.0030 (18)
Na3	0.0519 (14)	0.0519 (14)	0.0076 (13)	0.000	0.000	0.000
O1	0.0111 (11)	0.0152 (12)	0.0052 (10)	−0.0017 (10)	0.0014 (9)	0.0012 (8)
O2	0.0126 (10)	0.0086 (10)	0.0068 (9)	−0.0020 (10)	0.0019 (9)	0.0003 (9)
O3	0.0113 (12)	0.0106 (11)	0.0157 (11)	−0.0022 (10)	−0.0030 (9)	0.0052 (10)
O4	0.0121 (11)	0.0289 (14)	0.0031 (10)	0.0044 (11)	−0.0018 (9)	−0.0023 (9)
O5	0.0206 (13)	0.0092 (11)	0.0154 (12)	0.0006 (11)	−0.0005 (10)	−0.0008 (9)
O6	0.0120 (11)	0.0131 (11)	0.0137 (11)	0.0043 (9)	0.0025 (10)	0.0025 (10)
O7	0.0279 (13)	0.0099 (12)	0.0089 (10)	−0.0072 (11)	0.0060 (9)	−0.0029 (9)
O8	0.0088 (10)	0.0115 (10)	0.0090 (10)	0.0018 (9)	0.0002 (9)	0.0020 (9)

### Geometric parameters (Å, º)

**Table:**

Cr1—O6	1.965 (2)	Na1—O2	2.335 (2)
Cr1—O1^i^	1.967 (2)	Na1—O7	2.410 (3)
Cr1—O8	1.972 (2)	Na1—O5^vii^	2.455 (3)
Cr1—O3	1.973 (2)	Na1—O1^viii^	2.459 (3)
Cr1—O2	1.977 (2)	Na1—O3^vii^	2.621 (3)
Cr1—O4	1.993 (2)	Na1—O4^viii^	2.782 (3)
P1—O6^ii^	1.496 (2)	Na1—O3^viii^	2.826 (3)
P1—O1	1.515 (2)	Na1—O4	2.892 (3)
P1—O4	1.518 (2)	Na2—O5^ix^	2.254 (9)
P1—O7	1.593 (3)	Na2—O5^ii^	2.305 (9)
P2—O5	1.496 (2)	Na2—O5^x^	2.321 (10)
P2—O2^iii^	1.514 (2)	Na2—O5^xi^	2.366 (9)
P2—O3	1.523 (2)	Na2—O7^xii^	2.744 (9)
P2—O7^iv^	1.620 (2)	Na3—O8^viii^	2.542 (2)
P3—O8^iii^	1.545 (2)	Na3—O8^vi^	2.542 (2)
P3—O8^v^	1.545 (2)	Na3—O8	2.542 (2)
P3—O8	1.545 (2)	Na3—O8^iv^	2.542 (2)
P3—O8^vi^	1.545 (2)		
			
O6—Cr1—O1^i^	89.58 (10)	O2—Na1—O3^vii^	108.46 (9)
O6—Cr1—O8	173.94 (10)	O7—Na1—O3^vii^	122.20 (10)
O1^i^—Cr1—O8	93.87 (10)	O5^vii^—Na1—O3^vii^	58.61 (8)
O6—Cr1—O3	89.26 (10)	O1^viii^—Na1—O3^vii^	80.42 (8)
O1^i^—Cr1—O3	89.08 (10)	O2—Na1—O4^viii^	122.82 (9)
O8—Cr1—O3	95.77 (9)	O7—Na1—O4^viii^	100.16 (9)
O6—Cr1—O2	84.11 (9)	O5^vii^—Na1—O4^viii^	137.47 (10)
O1^i^—Cr1—O2	90.93 (9)	O1^viii^—Na1—O4^viii^	55.52 (7)
O8—Cr1—O2	90.84 (9)	O3^vii^—Na1—O4^viii^	92.97 (8)
O3—Cr1—O2	173.37 (10)	O2—Na1—O3^viii^	163.67 (10)
O6—Cr1—O4	93.21 (10)	O7—Na1—O3^viii^	55.20 (8)
O1^i^—Cr1—O4	176.41 (10)	O5^vii^—Na1—O3^viii^	86.26 (8)
O8—Cr1—O4	83.55 (9)	O1^viii^—Na1—O3^viii^	112.39 (8)
O3—Cr1—O4	88.70 (10)	O3^vii^—Na1—O3^viii^	87.08 (7)
O2—Cr1—O4	91.60 (10)	O4^viii^—Na1—O3^viii^	59.27 (7)
O6^ii^—P1—O1	115.82 (13)	O2—Na1—O4	64.97 (8)
O6^ii^—P1—O4	114.92 (14)	O7—Na1—O4	54.17 (8)
O1—P1—O4	108.24 (13)	O5^vii^—Na1—O4	108.96 (9)
O6^ii^—P1—O7	104.97 (13)	O1^viii^—Na1—O4	108.52 (9)
O1—P1—O7	107.96 (13)	O3^vii^—Na1—O4	166.12 (9)
O4—P1—O7	104.00 (14)	O4^viii^—Na1—O4	100.85 (10)
O5—P2—O2^iii^	114.64 (13)	O3^viii^—Na1—O4	98.76 (8)
O5—P2—O3	111.00 (14)	O5^ix^—Na2—O5^ii^	178.4 (3)
O2^iii^—P2—O3	113.01 (13)	O5^ix^—Na2—O5^x^	93.46 (16)
O5—P2—O7^iv^	105.71 (14)	O5^ii^—Na2—O5^x^	87.5 (4)
O2^iii^—P2—O7^iv^	108.91 (13)	O5^ix^—Na2—O5^xi^	87.6 (4)
O3—P2—O7^iv^	102.57 (13)	O5^ii^—Na2—O5^xi^	90.98 (15)
O8^iii^—P3—O8^v^	105.79 (16)	O5^x^—Na2—O5^xi^	153.1 (3)
O8^iii^—P3—O8	111.34 (8)	O5^ix^—Na2—O7^xii^	123.3 (2)
O8^v^—P3—O8	111.34 (8)	O5^ii^—Na2—O7^xii^	58.2 (2)
O8^iii^—P3—O8^vi^	111.34 (8)	O5^x^—Na2—O7^xii^	71.2 (3)
O8^v^—P3—O8^vi^	111.34 (8)	O5^xi^—Na2—O7^xii^	129.47 (19)
O8—P3—O8^vi^	105.79 (16)	O8^viii^—Na3—O8^vi^	139.91 (6)
O2—Na1—O7	110.26 (10)	O8^viii^—Na3—O8	139.91 (6)
O2—Na1—O5^vii^	97.43 (9)	O8^vi^—Na3—O8	57.99 (9)
O7—Na1—O5^vii^	75.20 (9)	O8^viii^—Na3—O8^iv^	57.99 (9)
O2—Na1—O1^viii^	76.17 (8)	O8^vi^—Na3—O8^iv^	139.91 (6)
O7—Na1—O1^viii^	149.73 (10)	O8—Na3—O8^iv^	139.91 (6)
O5^vii^—Na1—O1^viii^	134.49 (10)		

Symmetry codes: (i) *x*, *y*, *z*−1; (ii) *y*+1/2, *x*−1/2, *z*+1/2; (iii) *y*, −*x*+1, −*z*; (iv) *y*, −*x*+1, −*z*+1; (v) −*y*+1, *x*, −*z*; (vi) −*x*+1, −*y*+1, *z*; (vii) −*x*+3/2, *y*+1/2, −*z*+1/2; (viii) −*y*+1, *x*, −*z*+1; (ix) −*x*+1, −*y*, *z*+1; (x) *x*, *y*, *z*+1; (xi) −*y*+1/2, −*x*+1/2, *z*+1/2; (xii) −*x*+3/2, *y*−1/2, −*z*+3/2.
